# Higher Levels of Pre-operative Peripheral Lymphocyte Count Is a Favorable Prognostic Factor for Patients With Stage I and II Rectal Cancer

**DOI:** 10.3389/fonc.2019.00960

**Published:** 2019-09-24

**Authors:** Ying-Ying Zhang, Wan-Qing Li, Zhen-Fa Li, Xiao-Hua Guo, Shen-Kang Zhou, Aifen Lin, Wei-Hua Yan

**Affiliations:** ^1^School of Laboratory Medicine and Life Sciences, Wenzhou Medical University, Wenzhou, China; ^2^Department of Gastrointestinal Surgery, Taizhou Hospital of Zhejiang Province, Wenzhou Medical University, Linhai, China

**Keywords:** colorectal cancer, blood lymphocyte count, survival, prognosis, biomarker

## Abstract

The clinical significance of peripheral blood parameters has been considered to be a potential prognostic indicator for malignancies. In this study, 224 colorectal cancer (CRC; n_colon_ = 103; n_rectal_ = 121) patients who underwent resection were enrolled, and the pre- and post-operative clinical laboratory data within 1 week, before and after surgery, were collected. The prognostic value of the counts of white blood cell (WBC), neutrophil, lymphocyte and platelet, the neutrophil to lymphocyte ratio (NLR), and systemic immune-inflammation index (SII) were analyzed. Data revealed that pre-operative lymphocyte count (pre-LC) was much higher than that of post-LC (*p* < 0.001), and only rectal cancer patients with pre-LC^high^ (>median: 1.61 × 10^9^/L) had a significantly better overall survival (OS) and 5-year survival rate (SR) than those with pre-LC^low^ (OS: 62.3 vs. 49.5 months; SR: 74.0 vs. 43.0%; *p* = 0.006). Cox's proportional hazard models revealed that pre-LC^high^ was an independent, favorable prognostic factor for rectal cancer patients (hazard ratio = 0.348; *p* = 0.003). Moreover, when the disease stages were stratified, the pre-LC^high^ was significantly associated with better prognosis of rectal cancer patients with stage I + II rectal cancer (*n* = 65; OS: 67.5 vs. 54.3 months; *p* = 0.011). Taken together, our study revealed that pre-operative lymphocyte count is an independent prognostic factor for patients with stage I and II rectal cancer.

## Introduction

Peripheral lymphocytes, neutrophils, and monocytes in the complete blood cell count have been considered to play important roles in cellular-mediated inflammatory response in cancers (1). In this context, peripheral immune cell alterations have been frequently observed among various cancer patients, and their clinical significance, including the prognostic value of peripheral blood test parameters, have been intensively investigated during the past decades (2, 3). A large body of studies have revealed that both pre-operative and/or post-operative peripheral blood test parameters such as the counts of white blood cells (WBC), neutrophils, lymphocytes, monocytes and platelets, and derived neutrophil to lymphocyte ratio (NLR), platelet-to-lymphocyte ratio (PLR), lymphocyte-to-monocyte ratio (LMR), systemic immune-inflammation index (SII = count of platelet^*^neutrophil/lymphocyte), could be potential diagnostic and prognostic biomarkers for different types of cancers (4–6).

Colorectal cancer (CRC) ranks one of the leading cause of cancer-related death around the world (7). Although the tumor (T)-lymph nodes (N)-metastasis (M) TNM staging system has been widely used for CRC outcome prediction, the prognostic significance of peripheral blood test parameters have also been acknowledged in previous studies (8–10). However, due to the heterogeneity of the cohorts and different protocols of the study design such as the cut-off values, considerable discrepancy among the findings has been raised. Therefore, more studies are necessary to explore the predictive value of these peripheral systemic inflammation markers in CRC (11).

Previous reports revealed that PLR is an independent prognostic factor for survival in stage II CRC patients and in refractory metastatic CRC patients, where low PLR patients have a better prognosis (12, 13). In other cohorts of stage II and metastatic CRC patients, lower NLR, but not PLR is an independent favorable prognosis predictor (14, 15). An increasing number of both PLR factors and PLR associated with poor survival have been observed in CRC patients (16). Moreover, the prognostic factors, such as SII and MRR (monocyte to red blood cell count ratio), have been found to be superior to other factors including NLR, PLR, and LMR. Authors further strengthened that SII is of high prognostic stratification power in terms of particular TNM subgroups, and elevated MRR is associated with poor survival in CRC patients with early stages of rectal cancer (17, 18). Moreover, the clinical significance of the absolute count of WBC and its subsets have been explored in many studies. In this context, higher levels of pre-operative and/or post-operative lymphocyte count was found to be associated with better survival in CRC patients (19, 20).

In the current study, we retrospectively reviewed 224 CRC patients and evaluated the prognostic value of pre-operative and post-operative absolute count of WBC, neutrophils, lymphocytes, platelets and the derived NLR and SII in these CRC patients.

## Patients and Methods

### Patients

This study was reviewed and approved by the Institutional Ethics Review Board of Taizhou Hospital of Zhejiang Province, and written informed consent was obtained from each participant prior to the surgery.

We retrospectively reviewed 224 CRC (n_colon_ = 103; n_rectal_ = 121) patients who underwent surgical R0 curative resection at Taizhou Hospital of Zhejiang Province, Wenzhou Medical University between December 15th, 2010 and March 4th, 2013. Patients received pre-operative chemoradiation treatment or other medical interventions before surgery were excluded. Detail patient characteristics including age (median: 67 years; range: 39–89), gender, clinicopathological diagnosis, date of surgical operation, pre- and post- operative laboratory data within 1 week, were retrieved from the patient medical records provided by the Tissue Bank of Taizhou Hospital of Zhejiang Province (National Human Genetic Resources Platform of China YCZYPT [2017] 02). Tumor clinicopathologic stages were determined according to the 7th TNM staging system by the American Joint Committee for Cancer and International Union for Cancer Control (21). There were 74 patients with stage I, 48 patients with stage II, 98 patients with stage III, and 4 patients with stage IV, respectively.

Follow-up has documented for all patients until the December 31st 2016. The median follow-up was 48 months. The Overall survival was defined as the interval between the date of surgery and time of patient death (event) or the time of the last follow-up (censored).

### Laboratory Data

Routine laboratory data, including pre-operative and post-operative absolute count of WBC, neutrophils, lymphocytes, platelets from routine blood tests, were included in this study. Pre-operative and post-operative laboratory data were collected 1 week before and after surgery, respectively. The derived NLR and SII were calculated from the above data. NLR = count of neutrophils/lymphocytes. SII = count of platelet^*^neutrophil/lymphocyte (22).

### Statistical Analysis

All statistical analyses were performed with SPSS 13.0 (SPSS, Inc., Chicago, IL). Normality of continuous variables were analyzed by one-sample Kolmogorov-Smirnov test. Skewed distribution variables, including WBC and subsets count, platelet, NLR and SII between groups, were analyzed with Mann–Whitney's *U*-test. A correlation between pre- and post-operative lymphocyte count was analyzed using the Spearman method. Overall survival probabilities were analyzed using the Kaplan-Meier method. Differences between survival curves were analyzed by the log-rank test. The prognostic power of multiple clinicopathological variables were analyzed with univariate and multivariate Cox regression model. All statistical analysis were two-sided and *p* < 0.05 was considered statistically significant.

## Results

### Relationships Between Clinicopathologic Characteristics and Laboratory Variables

In this study, 224 CRC patients (n_colon_ = 103; n_rectal_ = 121) were enrolled, with 127 male and 97 female patients with a median age of 67 years (range: 30–89 years). Detail patient characteristics and their association between pre- and post-operative laboratory variables were listed in Table 1 and Supplementary Table 1, respectively.

**Table 1:** Pre-operation laboratory data and their association with clinicopathological parameters in CRC patients^*^.

**Variables**	**No**.	**WBC**		**Neutrophil**		**Lymphocyte**		**Platelet**		**NLR**		**SII**	
All CRC	224	6.75 (2.4–16.5)	*P*	4.23 (1.27–15.51)	*P*	1.61 (0.50–3.74)	*P*	243.0 (81–601)	*P*	2.60 (0.90–31.0)	*P*	642.2 (108.5–7072.6)	*P*
Colon	103	6.30 (2.4–14.3)	0.014	4.09 (1.27–11.50)	0.075	1.46 (0.63–3.74)	0.008	261.0 (81–601)	0.041	2.80 (0.90–18.3)	0.859	622.0 (132.7–4621.7)	0.420
Rectal	121	7.00 (3.3–16.5)		4.49 (1.49–15.51)		1.66 (0.50–3.45)		230.0 (114–494)		2.60 (0.90–31.0)		644.6 (108.5–7072.6)	
**Gender**													
Male	127	6.90 (2.4–16.5)	0.081	4.56 (1.37–15.51)	0.048	1.60 (0.50–3.74)	0.856	224.0 (81–601)	< .0.01	2.90 (1.20–31.0)	0.034	622.0 (132.7–7072.6)	0.553
Female	97	6.60 (2.70–15.7)		3.89 (1.27–12.32)		1.63 (0.51–3.45)		270.0 (93–586)		2.40 (0.90–11.0)		649.7 (108.5–4621.7)	
**Age**													
≤67 years	109	6.60 (3.30–14.3)	0.769	4.24 (1.49–11.50)	0.795	1.65 (0.63–3.74)	0.197	248.0 (81–601)	0.418	2.50 (1.10–18.3)	0.564	593.2 (132.7–4621.7)	0.582
>67 years	115	6.80 (2.40–16.5)		4.21 (1.27–15.51)		1.54 (0.50–3.45)		233.0 (110–458)		2.80 (0.90–31.0)		644.6 (108.5–7072.6)	
**T category**													
T2	110	6.75 (3.30–15.7)	0.590	4.34 (1.60–11.50)	0.634	1.66 (0.51–3.74)	0.100	238.5 (93–504)	0.261	2.40 (0.90–18.3)	0.332	606.5 (108.5–4271.4)	0.215
T3	108	6.60 (2.40–16.5)		4.10 (1.27–15.51)		1.46 (0.50–3.17)		245.0 (81–601)		2.70 (1.10–31.0)		654.4 (132.7–7072.6)	
T4	6	7.50 (5.20–8.70)		5.28 (3.38–5.92)		1.84 (1.04–1.90)		308.0 (216–363)		3.10 (2.50–3.40)		966.5 (591.7–1131)	
**N category**													
N0	125	6.90 (2.70–15.8)	0.722	4.53 (1.27–14.1)	0.466	1.60 (0.51–3.74)	0.068	243.0 (93–504)	0.617	2.60 (0.90–14.8)	0.048	659.1 (108.5–4621.7)	0.304
N1	56	6.55 (2.40–16.5)		4.00 (1.37–15.5)		1.46 (0.50–3.45)		231.5 (113–420)		2.85 (1.10–31.0)		646.8 (169.5–7072.6)	
N2	43	6.40 (3.30–12.7)		3.70 (1.49–11.5)		1.70 (0.63–3.17)		249.0 (81–601)		2.10 (1.10–18.3)		584.2 (132.7–4271.4)	
**M category**													
M0	220	6.65 (2.40–16.5)	0.503	4.23 (1.27–15.51)	0.544	1.61 (0.50–3.74)	0.867	239.5 (81–601)	0.016	2.60 (0.90–31.0)	0.549	635.2 (108.5–7072.6)	0.135
M1	4	7.75 (5.20–8.70)		4.83 (3.38–7.57)		1.64 (0.76–2.35)		375.0 (262–464)		2.85 (1.80–10.0)		923.6 (631–4621.7)	
**AJCC stage**													
I	74	6.80 (3.30–12.9)	0.774	4.36 (1.60–9.35)	0.493	1.64 (0.51–3.74)	0.517	235.0 (93–504)	0.108	2.40 (0.90–9.40)	0.149	587.4 (108.5–2852.2)	0.131
II	48	6.90 (2.70–15.8)		4.62 (1.27–14.1)		1.43 (0.74–2.75)		252.5 (110–483)		3.00 (1.10–14.8)		764.0 (155.6–4580.1)	
III	98	6.50 (2.40–16.5)		3.97 (1.37–15.5)		1.64 (0.50–3.45)		236.5 (81–601)		2.55 (1.10–31.0)		606.5 (132.7–7072.6)	
IV	4	7.75 (5.20–8.70)		4.83 (3.38–7.57)		1.64 (0.76–2.35)		375.0 (262–464)		2.85 (1.80–10.0)		923.6 (631–4621.7)	
**Patient status**													
Survival	160	6.75 (2.40–15.8)	0.413	4.24 (1.37–14.1)	0.982	1.66 (0.63–3.74)	0.029	244.0 (96–601)	0.183	2.50 (0.90–18.3)	0.173	616.4 (108.5–4580.1)	0.638
Dead	64	6.70 (2.70–16.5)		4.23 (1.27–15.5)		1.42 (0.50–3.17)		232.5 (81–464)		2.80 (1.00–31.0)		646.8 (132.7–7072.6)	

* *Laboratory variables presented as median and range; WBC, Neutrophil, Lymphocyte and Platelet (10^9^/L); Mann–Whitney U-test was applied for the comparison of each variable*.

Data showed that only the pre-operative lymphocyte count (pre-LC) were significantly associated with the status of patient survival, that the pre-LC was much higher in survived patients than in the CRC patients who died (1.66 × 10^9^/L vs. 1.42 × 10^9^/L; *p* = 0.029; Table 1; Supplementary Table 1). Moreover, we found that pre-LC was significantly higher than post-operative lymphocyte count (post-LC) for CRC patients (1.61 × 10^9^/L vs. 1.10 × 10^9^/L; *p* < 0.001; Supplementary Figure 1A), and the levels between pre- and post-LC were significantly correlated (R = 0.502; *p* < 0.001; Supplementary Figure 1B). Moreover, data revealed that both pre- and post-LC were significantly higher in rectal cancer patients than in colon cancer patients (Table 1; Supplementary Table 1).

### Pre-operative Lymphocyte Count Related to CRC Patient Survival

**Figure 1:**
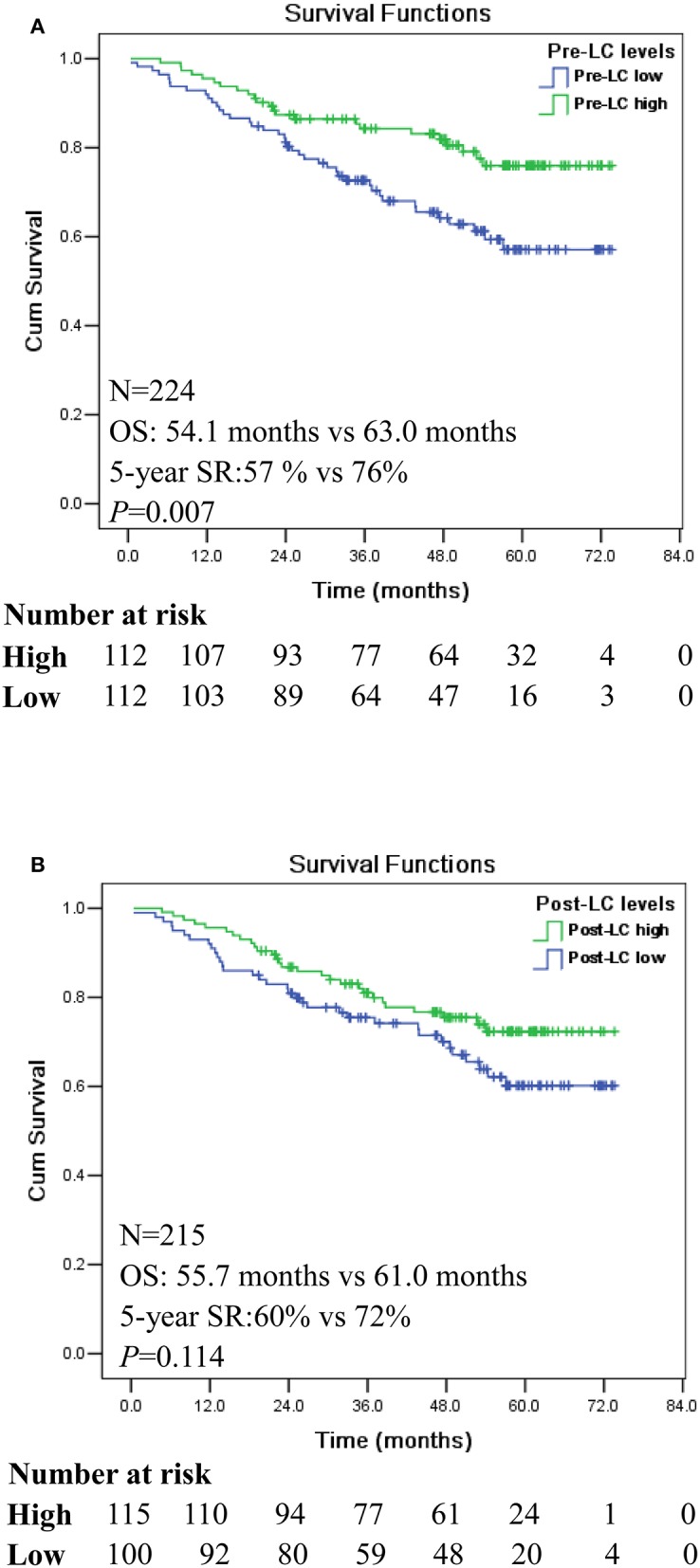
Kaplan-Meier survival analysis between the high and low levels of pre- and post-operation peripheral lymphocyte count (LC) in CRC patients. **(A)** Comparison of overall survival between pre-LC^high^ and pre-LC^low^ among CRC patients. **(B)** Comparison of overall survival between post-LC^high^ and post-LC^low^ among CRC patients.

CRC patients were classified as pre-LC^high^ (*n* = 112) and pre-LC^low^ (*n* = 112) groups, respectively, according to the median level of lymphocyte count (median: 1.61 × 10^9^/L). Kaplan-Meier analysis showed that patients with pre-LC^high^ had significantly longer survival [mean: 63 (95% CI: 59.0–67.0) vs. 54.1 months (95% CI: 49.3–59.0)] and 5-year survival rate (SR: 76 vs. 57%) than that of patients with pre-LC^low^ (*p* = 0.007; Figure 1A), respectively. However, no significant difference was observed between the patients with post-LC^high^ (*n* = 115) and post-LC^low^ [*n* = 110; mean: 61.0 (95% CI: 56.9–65.2) vs. 55.7 months (95% CI: 50.7–60.8); *p* = 0.114; Figure 1B].

### Prognostic Stratification of Pre-operative Lymphocyte Count in CRC Patients

The prognostic stratification value of peripheral blood variables, such as high platelet count, have been found to be independent prognostic predictors only in patients with stage IV CRC patients (23). Other studies also revealed that SII was of prognostic stratification power for each disease stage (17), and elevated MRR was associated with poor survival in CRC patients with early disease stages I and II (18).

**Figure 2:**
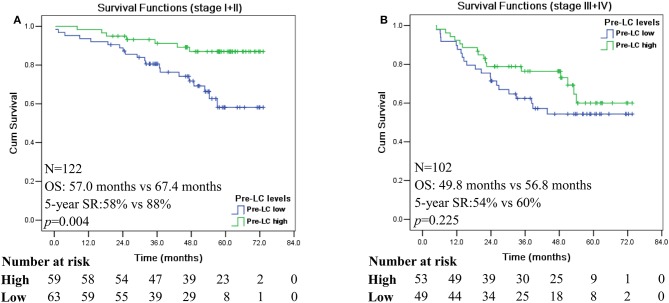
Kaplan-Meier survival analysis of the high and low levels of pre-operation peripheral lymphocyte count in different disease stage of CRC patients. Comparison of overall survival between pre-LC^high^ and pre-LC^low^ among CRC patients with disease stage I + II **(A)** and disease stage III + IV **(B)**.

In this study, our data showed that pre-LC^high^ is associated with a significantly better survival in CRC patients with early disease stage of AJCC I and II (*n* = 122; *p* = 0.004; Figure 2A), whereas no significant difference was reached for patients with advanced disease stage III and IV (*n* = 102; *p* = 0.225; Figure 2B). Among disease stage I and II, patients with pre-LC^high^ had significantly longer survival (mean: 67.4 vs. 57.0 months) and 5-year SR (88 vs. 58%) than that of patients with pre-LC^low^, respectively (Figure 2A), while no significance was reached for the survival of CRC patients with stage III and IV between the pre-LC^low^ and pre-LC^high^ (mean: 49.8 vs. 56.8 months) and 5-year SR (54 vs. 60%; Figure 2B).

**Figure 3:**
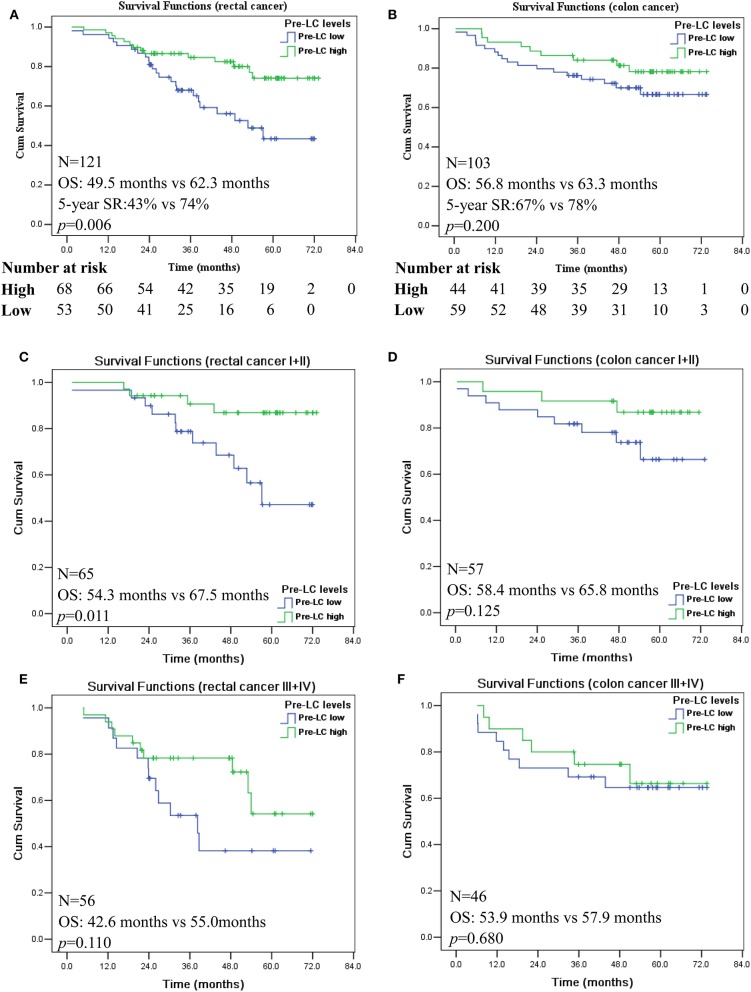
Kaplan-Meier survival analysis of the high and low levels of pre-operation peripheral lymphocyte count in rectal and colon cancer patients with AJCC I + II and III + IV disease stages. Comparison of overall survival between pre-LC^high^ and pre-LC^low^ among patients with rectal cancer **(A)**; colon cancer **(B)**; rectal cancer patients with disease stage I + II **(C)** and III + IV **(E)**; colon cancer patients with disease stage I + II **(D)** and III + IV **(F)**.

Next, we analyzed the prognostic significance of pre-LC with stratification of colon and rectal cancer, respectively. A survival curve revealed that pre-operative LC^high^ and LC^low^ is of prognostic stratification value only in patients with rectal cancer (*n* = 121; *p* = 0.006; Figure 3A), but not in patients with colon cancer (*n* = 103; *p* = 0.200; Figure 3B). Moreover, when the disease stages were stratified, the pre-LC^high^ was only significantly associated with better prognosis of rectal cancer patients with stages I + II (*n* = 65; *p* = 0.011; Figure 3C), while no prognostic significance of the status of pre-LC^high^ and pre-LC^low^ was observed for the rectal cancer patients with stages III + IV (*n* = 56; *p* = 0.110; Figure 3E), as well as colon cancer patients with stages I + II (*n* = 57; *p* = 0.125; Figure 3D) and III + IV (*n* = 46; *p* = 0.680; Figure 3F).

### Prognosis Value of Pre-operative Lymphocyte Count for Patients With Rectal Cancer

Finally, the prognostic value of pre-LC for patients with rectal cancer was assessed with the Cox's proportional hazards model. Univariate analysis data showed that pathological parameters including patient age (HR = 2.067, *p* = 0.036), pM (HR = 4.907, *p* = 0.030), AJCC stage (HR = 2.278, *p* = 0.015), and the pre-LC (LC^high^ vs. LC^low^, HR = 0.403, *p* = 0.008) were dramatically associated to the prognosis of patients with rectal cancer. In addition to the AJCC disease stages (HR = 2.399, *p* = 0.012), multivariate analysis showed that pre- LC^high^ vs. LC^low^ remains statistically significant and can be an independent prognostic factor (HR = 0.348, *p* = 0.003), indicating that pre-LC^high^ is an favorable predictor for patients with rectal cancer (Table 2).

**Table 2:** Cox proportional hazards model for variables affecting overall survival in rectal cancer patients.

**Variables**	**Categories**	**Univariate**		**Multivariate**	
		**HR (95% CI)**	***P***	**HR (95% CI)**	***P***
Gender	Male vs. female	0.991 (0.497–1.973)	0.979	/	
Age (years)	>67 vs. ≤67	2.067 (1.049–4.073)	0.036	1.949 (0.974–3.903)	0.059
T category	T_3+4_ vs. T_2_	1.398 (0.733–2.664)	0.309	/	
N category	N_1+2_ vs. N_0_	1.404 (0.774–2.545)	0.264	/	
M category	M_1_ vs. M_0_	4.907 (1.169–20.60)	0.030	4.146 (0.827–19.71)	0.074
AJCC stage	III/IV vs. I/II	2.278 (1.176–4.413)	0.015	2.399 (1.216–4.730)	0.012
WBC (10^9^/L)	High vs. low^*^ (median)	1.021 (0.532–1.960)	0.950	/	
Neutrophil (10^9^/L)		0.866 (0.449–1.671)	0.668	/	
Lymphocyte (10^9^/L)		0.403 (0.207–0.786)	0.008	0.348 (0.172–0.705)	0.003
Platelet (10^9^/L)		0.696 (0.365–1.327)	0.272	/	
NLR		0.724 (0.376–1.396)	0.335	/	
SII		0.678 (0.352–1.308)	0.246	/	

*HR, hazard ratio; 95% CI, 95% confidence interval*.

* *Pre-operation laboratory data*.

## Discussion

In addition to widely used risk factors, including tumor itself features (TNM and AJCC stages, histopathological types, etc.) and patient individual characteristics (gender, age, immunological status, genetic background, etc.), the prognostic value of the peripheral hematological markers, such as WBC and subset count and their derived parameters, such as SII, NLR, MLR, and MRR, has suggested this to be a promising field that has been carried out in many malignancies (11, 24, 25).

In this study, we found that only the higher pre-operative lymphocyte count (above the median level), is an independent favorable prognostic predictor for patients with stage I and II rectal cancer. However, no prognostic significance of pre-operative lymphocyte count was observed for patients with rectal cancer stage III and IV, and for patients with colon cancer. In previous studies, the prognostic significance of peripheral hematological markers and their derived parameters PLR, NLR, or SII have been investigated. However, conclusions remain discrepant. Among these studies, different prognostic significance of PLR and NLR have been found in CRC patients with disease stage II and in refractory metastatic CRC patients (13, 14). Chen et al. (17) reported that SII was an independent risk factor for both overall survival and disease-free survival; however, in another study, SII was a risk factor only for the disease-free survival (26). The discrepancy of findings might be due to the heterogeneity of the cohorts and different protocols of the study design, such as the cut-off values, or even the genetic background of a particular patient. In this context, NLR has been found of prognostic value only in mismatch repair-proficient (pMMR), but not in CRC patients with mismatch repair-deficient (dMMR) (27). Other factors like pre-operative circulating eosinophil and basophil count have been reported to affect the predictive value of the PLR and NLR in CRC patients with stage I–III (28).

In the current study, our data revealed a strong correlation between the pre-operative LC and post-operative LC, while the levels of post-operative LC were dramatically decreased. Our findings also showed that only the higher levels of pre-operative LC were significantly associated with a better survival and an independent predictive factor for patients with stage I and II rectal cancer. A disease stage dependent predictive value of other parameters, such as NLR, LMR, and MRR, have been observed in previous studies. However, the immunological mechanism for this phenomenon remains to be explored. In this context, LMR has been found to be a favorable prognostic factor for early stage of CRC; however, LMR and MRR have a better prognostic effect on advanced stage rather than early stage of CRC (18, 29, 30). Though the prognostic significance of either pre- or post-operative LC has been investigated, studies comparison between pre- or postoperative LC are less available (31–35). A strong correlation between pre-operative LC and post-operative LC in CRC patients had been reported in a previous study by Yamamoto et al. (19), and the study also showed that the post-operative lymphocyte count was dramatically lower than the pre-operative lymphocyte count. However, in that study, the authors found that both pre-operative and post-operative LC were related to prognosis of CRC patients, and the combination of pre- and post-operative LC had a more powerful prognostic value than either pre- or post-operative LC alone. In other studies, Iseki et al. (31) investigated the prognostic value of count and percentage of pre-operative peripheral lymphocyte in 362 patients with colorectal cancer. With the cut-off of 1.7 × 10^9^/L for pre-operative lymphocyte count, authors found that the 5-year relapse-free survival (RFS) and overall survival (OS) rate in the high lymphocyte count group was significantly higher than that in the low lymphocyte count group. In line with this, another study with the cut-off of 1.0 × 10^9^/L for pre-operative lymphocyte count, Shibutani et al. (32) showed that RFS and OS rates were significantly higher in the high-lymphocyte group compared with the low-lymphocyte group. On the contrary, no prognostic significance of pre-operative lymphocyte count was observed for both RFS and OS in another cohort of patients with colorectal cancer (33). The discrepancy findings between previous studies might be owing to the different cut-off values, and actually no reference of cut-off values has been recommended or established (36). Other factors can also affect the predictive value of laboratory markers, such as heterogeneous cohorts of CRC patients, by simultaneous inclusion of colon and rectal cancer patients, and by different treatment options especially in the administration of different adjuvant chemotherapy regimens or not (37).

In summary, our findings revealed that pre-operative lymphocyte count is an independent prognostic factor for patients with stage I and II rectal cancer. However, the limited size of the cohort and the fact that only colorectal cancer patients who underwent radical resection were included in this retrospective study, are the main limitations. Therefore, larger prospective cohorts with a multicenter are necessary to validate the prognostic values of these non-invasive, cost-effective, and convenient systemic inflammatory indices.

## Data Availability Statement

All datasets generated for this study are included in the manuscript/Supplementary Files.

## Ethics Statement

This study was reviewed and approved by the Institutional Ethic Committee and Review Board of Taizhou Hospital of Zhejiang Province, written informed consent was obtained from each participant prior to the surgery.

## Author Contributions

AL and W-HY: study design, statistical analysis, and manuscript draft. Y-YZ, W-QL, Z-FL, X-HG, and S-KZ: clinical data review and acquisition. All authors read and approved the final manuscript.

### Conflict of Interest

The authors declare that the research was conducted in the absence of any commercial or financial relationships that could be construed as a potential conflict of interest.

## Supplementary Material

Supplementary Figure 1 **(A)** Comparison of levels between pre-operation (*n* = 224) and post-operation (*n* = 215) peripheral lymphocyte count in CRC patients with Mann–Whitney *U-*test. Blue bar represents the median. **(B)** Correlation between pre-operative and post-operative lymphocyte count with the Spearman method (*n* = 215).

Supplementary Table 1 Post-operation laboratory data and their association with clinicopathological parameters in CRC patients^*^.
