# Supplementary figures and images for: Higher Levels of Pre-operative Peripheral Lymphocyte Count Is a Favorable Prognostic Factor for Patients With Stage I and II Rectal Cancer
Source: Front Oncol. 2019 Sep 24;9:960. doi: 10.3389/fonc.2019.00960 (PMC6769073; doi:10.3389/fonc.2019.00960)

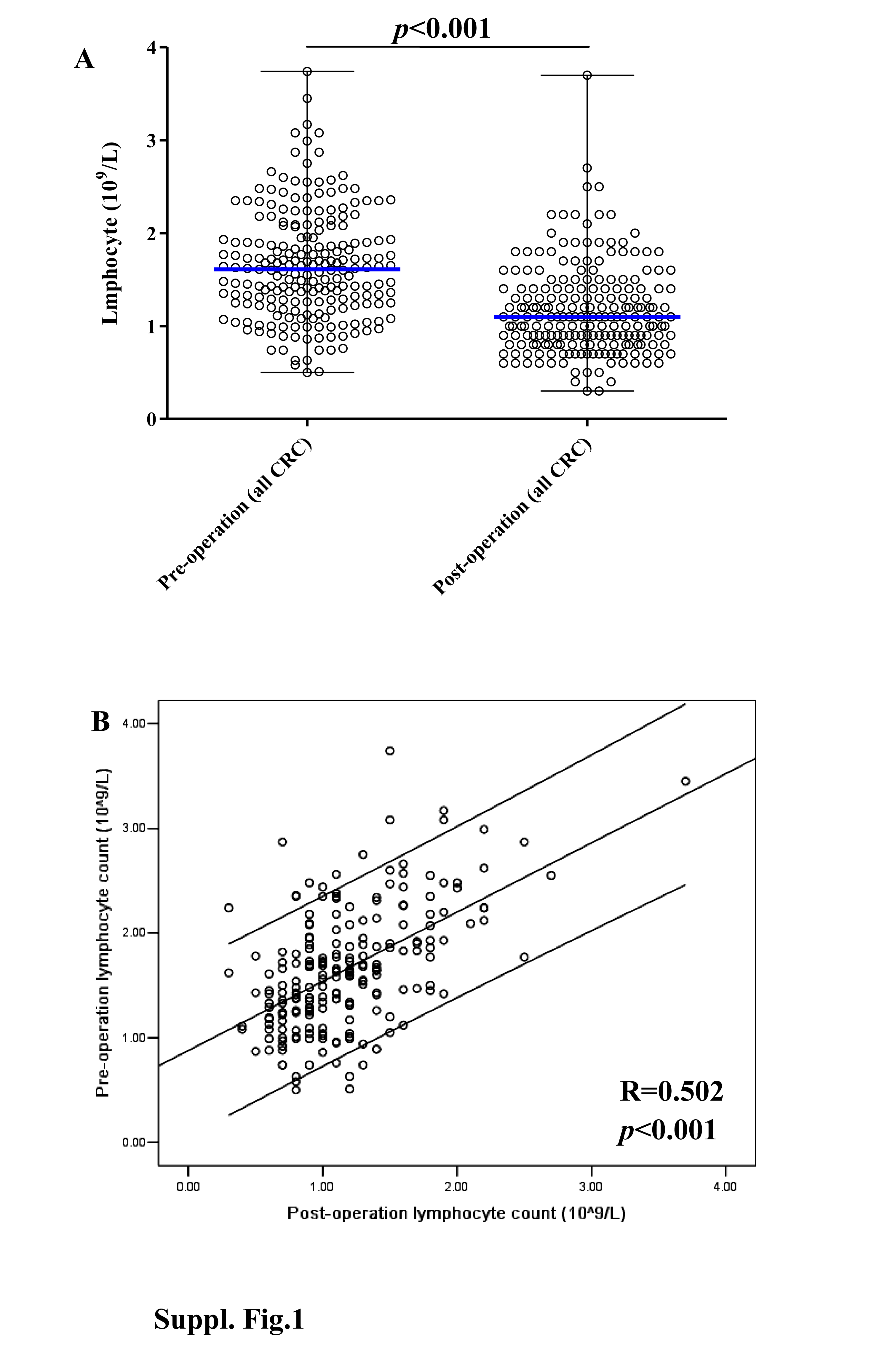

Supplement: Supplementary Figure 1 — (A) Comparison of levels between pre-operation (n = 224) and post-operation (n = 215) peripheral lymphocyte count in CRC patients with Mann–Whitney U-test. Blue bar represents the median. (B) Correlation between pre-operative and post-operative lymphocyte count with the Spearman method (n = 215). [file Image_1.TIF]
