# Supplementary material for: Higher Levels of Pre-operative Peripheral Lymphocyte Count Is a Favorable Prognostic Factor for Patients With Stage I and II Rectal Cancer
Source: Front Oncol. 2019 Sep 24;9:960. doi: 10.3389/fonc.2019.00960 (PMC6769073; doi:10.3389/fonc.2019.00960)
Supplement: Supplementary Table 1 — Post-operation laboratory data and their association with clinicopathological parameters in CRC patients*. [file Table_1.DOC]

| **Suppl. Table 1** Post-operation laboratory data and their association with clinicopathological parameters in CRC patients* | | | | | | | | | | | | | |
| --- | --- | --- | --- | --- | --- | --- | --- | --- | --- | --- | --- | --- | --- |
| Variables | No. | WBC |  | Neutrophil |  | Lymphocyte |  | Platelet |  | NLR |  | SII |  |
| All CRC | 215 | 7.10 (3.2-22.8) | *P* | 5.20 (2.10-21.70) | *P* | 1.10 (0.30-3.70) | *P* | 252.0 (89-544) | *P* | 5.04 (1.13-45.9) | *P* | 536.8 (109.1-4442.3) | *P* |
| colon | 100 | 6.55 (3.2-22.8) | <0.01 | 4.75 (2.20-21.70) | 0.002 | 1.00 (0.30-2.58) | 0.001 | 256.5 (89-544) | 0.452 | 4.65 (1.53-32.3) | 0.761 | 499.2 (150.8-3344.6) | 0.035 |
| rectal | 115 | 7.90 (3.3-17.6) |  | 5.80 (2.10-14.30) |  | 1.20 (0.30-3.70) |  | 246.0 (100-433) |  | 5.35 (1.13-45.9) |  | 595.5 (109.1-4442.3) |  |
| Gender |  |  |  |  |  |  |  |  |  |  |  |  |  |
| male | 121 | 7.10 (3.2-17.6) | 0.657 | 5.20 (2.10-14.30) | 0.692 | 1.10 (0.50-2.70) | 0.753 | 246.0 (89-544) | 0.117 | 5.33 (1.13-31.1) | 0.553 | 587.8 (154.2-4442.3) | 0.020 |
| female | 94 | 6.80 (3.2-22.8) |  | 5.20 (2.40-21.70) |  | 1.10 (0.30-3.70) |  | 261.0 (96-515) |  | 4.70 (1.23-45.9) |  | 475.6 (109.1-4087.4) |  |
| Age |  |  |  |  |  |  |  |  |  |  |  |  |  |
| ≤67 yrs | 107 | 7.10 (4.00-17.6) | 0.883 | 5.10 (2.50-14.30) | 0.875 | 1.20 (0.50-2.50) | 0.054 | 260.0 (89-544) | 0.098 | 4.64 (1.13-31.1) | 0.369 | 527.2 (150.9-4442.3) | 0.696 |
| >67 yrs | 108 | 6.90 (3.20-22.8) |  | 5.20 (2.10-21.70) |  | 1.00 (0.30-3.70) |  | 246.0 (96-506) |  | 5.69 (1.23-45.9) |  | 560.5 (109.1-4087.4) |  |
| T category |  |  |  |  |  |  |  |  |  |  |  |  |  |
| T2 | 103 | 7.20 (3.20-17.6) | 0.436 | 5.20 (2.20-13.60) | 0.709 | 1.10 (0.30-3.70) | 0.085 | 252.0 (119-447) | 0.171 | 4.64 (1.23-45.9) | 0.539 | 539.8 (109.1-4087.4) | 0.873 |
| T3 | 106 | 6.85 (3.20-22.8) |  | 5.20 (2.10-21.70) |  | 1.00 (0.30-2.20) |  | 251.0 (89-544) |  | 5.39(1.13-32.3) |  | 535.0 (150.1-4442.3) |  |
| T4 | 6 | 8.20 (4.80-12.7) |  | 6.00 (2.90-10.40) |  | 1.25 (0.90-1.60) |  | 322.0 (198-365) |  | 5.31 (2.66-7.48) |  | 532.5 (311.7-849.2) |  |
| N category |  |  |  |  |  |  |  |  |  |  |  |  |  |
| N0 | 118 | 7.20 (3.20-17.6) | 0.203 | 5.25 (2.50-14.3) | 0.120 | 1.10 (0.30-2.70) | 0.445 | 256.0 (96-477) | 0.496 | 5.36 (1.60-45.9) | 0.141 | 553.3 (181.8-4442.3) | 0.307 |
| N1 | 55 | 7.10 (3.20-22.8) |  | 5.30 (2.10-21.7) |  | 1.00 (0.40-3.70) |  | 246.0 (100-544) |  | 5.20 (1.59-31.9) |  | 539.8 (109.1-3344.6) |  |
| N2 | 42 | 6.50 (3.60-14.1) |  | 4.75 (2.40-10.9) |  | 1.10 (0.70-2.20) |  | 245.0 (89-515) |  | 4.27 (1.13-12.6) |  | 505.2 (154.2-1077.3) |  |
| M category |  |  |  |  |  |  |  |  |  |  |  |  |  |
| M0 | 211 | 6.90 (3.20-22.8) | 0.076 | 5.20 (2.10-21.7) | 0.046 | 1.10 (0.30-3.70) | 0.101 | 252.0 (89-544) | 0.198 | 4.93 (1.13-32.3) | 0.016 | 533.3 (109.1-4442.3) | 0.084 |
| M1 | 4 | 9.50 (7.20-13.6) |  | 7.70 (5.50-12.4) |  | 0.85 (0.30-1.10) |  | 282.0 (237-373) |  | 8.38 (6.44-45.9) |  | 827.9 (531.3-4087.4) |  |
| AJCC stage |  |  |  |  |  |  |  |  |  |  |  |  |  |
| I | 68 | 7.15 (3.90-17.6) | 0.170 | 5.10 (2.50-13.6) | 0.085 | 1.15 (0.50-2.70) | 0.052 | 255.5 (126-477) | 0.512 | 4.57 (1.60-22.8) | 0.010 | 518.3 (181.8-2739.2) | 0.138 |
| II | 47 | 6.90 (3.20-15.2) |  | 5.60 (2.50-14.3) |  | 1.10 (0.30-1.90) |  | 256.0 (96-419) |  | 6.02 (2.39-32.3) |  | 572.5 (223.2-4442.3) |  |
| III | 96 | 6.90 (3.20-22.8) |  | 4.90 (2.10-21.7) |  | 1.10 (0.40-3.70) |  | 246.0 (89-544) |  | 4.62 (1.13-31.9) |  | 524.8 (109.1-3344.6) |  |
| IV | 4 | 9.50 (7.20-13.6) |  | 7.70 (5.50-12.4) |  | 0.85 (0.30-1.10) |  | 282.0 (237-373) |  | 8.38 (6.44-45.9) |  | 827.9 (531.3-4087.4) |  |
| Patient status |  |  |  |  |  |  |  |  |  |  |  |  |  |
| survival | 153 | 6.90 (3.90-15.4) | 0.843 | 5.20 (2.40-14.3) | 0.509 | 1.10 (0.30-3.70) | 0.130 | 256.0 (110-515) | 0.565 | 4.70 (1.13-32.3) | 0.110 | 531.8 (109.1-4442.3) | 0.339 |
| dead | 62 | 7.30 (3.20-22.8) |  | 5.30 (2.10-21.7) |  | 1.00 (0.30-2.20) |  | 251.0 (89-544) |  | 5.60 (1.72-45.9) |  | 578.1 (201.1-4087.4) |  |
| *Laboratory variables presented as median and range; WBC, Neutrophil, Lymphocyte and Platelet (10^9^/L); Mann-Whitney U test was applied for the comparison of each variable. | | | | | | | | | | | | | |
